# Monitoring online media reports for early detection of unknown diseases: Insight from a retrospective study of COVID‐19 emergence

**DOI:** 10.1111/tbed.13738

**Published:** 2020-08-02

**Authors:** Sarah Valentin, Alizé Mercier, Renaud Lancelot, Mathieu Roche, Elena Arsevska

**Affiliations:** ^1^ UMR TETIS CIRAD Montpellier France; ^2^ TETIS AgroParisTech CIRAD CNRS INRAE Univ Montpellier Montpellier France; ^3^ UMR ASTRE CIRAD Montpellier France; ^4^ ASTRE CIRAD INRAE Univ Montpellier Montpellier France

**Keywords:** COVID‐19, emerging disease, epidemic intelligence, one Health, online news, PADI‐web

## Abstract

Event‐based surveillance (EBS) systems monitor a broad range of information sources to detect early signals of disease emergence, including new and unknown diseases. In December 2019, a newly identified coronavirus emerged in Wuhan (China), causing a global coronavirus disease (COVID‐19) pandemic. A retrospective study was conducted to evaluate the capacity of three event‐based surveillance (EBS) systems (ProMED, HealthMap and PADI‐web) to detect early COVID‐19 emergence signals. We focused on changes in online news vocabulary over the period before/after the identification of COVID‐19, while also assessing its contagiousness and pandemic potential. ProMED was the timeliest EBS, detecting signals one day before the official notification. At this early stage, the specific vocabulary used was related to ‘pneumonia symptoms’ and ‘mystery illness’. Once COVID‐19 was identified, the vocabulary changed to virus family and specific COVID‐19 acronyms. Our results suggest that the three EBS systems are complementary regarding data sources, and all require timeliness improvements. EBS methods should be adapted to the different stages of disease emergence to enhance early detection of future unknown disease outbreaks.

## 1 INTRODUCTION

Epidemic intelligence (EI) aims to detect, monitor and assess potential health threats for early warning and rapid response (Paquet, Coulombier, Kaiser, & Ciotti, 2006). In addition to indicator‐based surveillance of official sources, public and animal health agencies increasingly incorporate an event‐based surveillance (EBS) component into their EI system. EBS uses unstructured data from unofficial sources such as online news to improve early detection of emerging infectious diseases (EIDs). Several free‐access EBS systems have been supporting epidemic intelligence since the late 1990s, such as the Program for Monitoring Emerging Diseases (ProMED; Woodall, 2001), HealthMap (Brownstein, Freifeld, Reis, & Mandl, 2008) and the recently developed PADI‐web (Valentin, Arsevska, et al., 2020).

Program for Monitoring Emerging Diseases is a human‐curated system that was launched by the International Society for Infectious Diseases (ISID) in 1994. The system relies on a large network of experts worldwide who produce and share verified reports on disease outbreaks in a common platform (Carrion & Madoff, 2017). HealthMap is a semi‐automated system launched by the Boston Children's Hospital in 2006. The tool monitors both official and non‐official web news sources (Freifeld, Mandl, Reis, & Brownstein, 2008). Both HealthMap and ProMED monitor a list of human and animal diseases and syndromes thereof. The Platform for Automated extraction of animal Disease Information from the web (PADI‐web) was created in 2016 to monitor online animal health‐related news for the French Epidemic Intelligence System (FEIS) (Arsevska et al., 2018; Valentin, Arsevska, et al., 2020). Both HealthMap and PADI‐web automatically retrieve health‐related news from Google News using customized Really Simple Syndication (RSS) feeds. For news detection, the two systems mine terms for known diseases, as well as for clinical signs and syndromes (Arsevska et al., 2016). All three EBS systems monitor news in multiple languages, including Chinese.

On 31 December 2019, local health officials of the Chinese city of Wuhan reported a cluster of 27 cases of ‘pneumonia of unknown cause’. These cases were linked to a wholesale live animal and seafood market in the city. The first death was reported in January 2020, and the causative agent was identified as a new coronavirus, that is SARS‐CoV‐2, and the disease was named COVID‐19. The first epidemiological study on patients with laboratory‐confirmed COVID‐19 infection reported the onset of illness as early as 1 December 2019 (Huang et al., 2020).

This retrospective study aimed first to evaluate three EBS systems (ProMED, HealthMap and PADI‐web) and their capacity for timely detection of the COVID‐19 emergence in China. Secondly, we focused on PADI‐web to understand how an animal health EBS system contributed to the detection of a human EID. We analysed the RSS feeds from PADI‐web that detected COVID‐19‐related news articles (hereafter referred to as ‘news’). Thirdly, we assessed the vocabulary in the news detected by PADI‐web and its change in relation to identification of the pathogen and the EID spread.

## 2 MATERIAL AND METHODS

### 2.1 COVID‐19‐related news detection

News from 1 to 31 December 2019 was mined to assess the timeliness of the three EBS. We compared the first news regarding the publication date, language and source.

To gain insight into how PADI‐web detected the COVID‐19 emergence, we further filtered a second corpus of news published from 31 December 2019 to 26 January 2020 containing at least one of the following words in the title and body of the news: ‘pneumonia’, ‘respiratory illness’, ‘coronavirus’, ‘nCoV’ (an early name for COVID‐19), and ‘Wuhan’. After manual verification of their relevance, we retained 275 out of 333 news items for analysis (Valentin, Mercier, Mercier, Lancelot, Roche, & Arsevska, 2020).

We assessed the link between the detected news items and the animal health RSS feeds from PADI‐web that served to retrieve those news items. To this end, we read each news item and categorized it into (i) disease‐specific RSS feeds (containing specific disease names) and (ii) syndromic RSS feeds (containing combinations of symptoms and animal hosts).

### 2.2 News vocabulary

**Figure 1:**
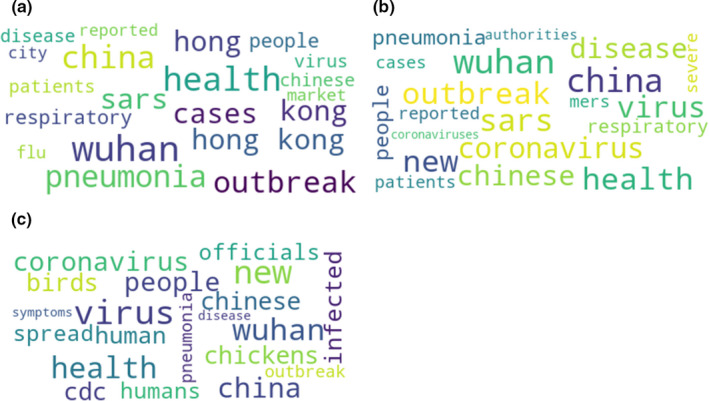
Wordclouds generated from COVID‐19‐related news articles during three consecutive periods: (a) 31 December 2019 – 08 January 2020, (b) 09–19 January 2020, (c) 20–26 January 2020 [Colour figure can be viewed at wileyonlinelibrary.com]

We analysed the vocabulary change spanning the period from the initial discovery of the COVID‐19 outbreak to its spread outside China by extracting terms from the whole corpus. A word frequency‐based method was first implemented to highlight important keywords according periods (Figure 1). Secondly, we used a ranking function based on the frequency and discriminance^1^ of terms (i.e. words and multi‐word terms) extracted with BioTex, a text‐mining tool tailored for biomedical terminology (Lossio‐Ventura, Jonquet, Roche, & Teisseire, 2016). BioTex is based on the use of (i) a relevant combination of information retrieval techniques and statistical methods and (ii) a list of syntactic structures of terms that have been learnt via relevant sources (e.g. UMLS, MeSH). BioTex‐extracted terms can be lowercase words (e.g. influenza), or phrases (e.g. avian influenza).

We further identified terms referring to COVID‐19, such as ‘new virus’ and ‘mystery pneumonia’. We manually categorized the terms as ‘mystery’ (referring to the unknown threat), ‘pneumonia’ (referring to the clinical signs), ‘coronavirus’ (referring to the virus taxonomy) and ‘technical’ (technical acronyms specifically pertaining to the virus). One news item could contain terms from different categories. We calculated the daily proportion of each category, expressed as the sum of occurrences of the category divided by the total number of occurrences.

## 3 RESULTS AND DISCUSSION

### 3.1 News detection

Program for Monitoring Emerging Diseases was the first EBS system to detect and report a news item from a Chinese online source.^2^ The ProMED report dated back to 30 December 2019—a day before the first official notification of pneumonia‐like cases in Wuhan (Wuhan Municipal Health Commission, 2020). PADI‐web and HealthMap respectively detected three and one COVID‐19‐related news items on 31 December 2019—the same day as the first official notification of pneumonia‐like cases in Wuhan (one HealthMap news item from an English source, three PADI‐web news items from two English sources and one Chinese source). The news detected by the three EBS originated from five different media outlets.

Among the three EBS systems compared, only ProMED relies on local expert information to alert on health threats. This result suggests that the network of local field experts is crucial for the detection of EID events and their reporting. Otherwise, HealthMap and PADI‐web detected news on the same day as the official reporting. It is therefore essential to understand their current limitations and promote the key role of experts in EBS systems. Further studies should also focus on assessing whether the timeliness of automated systems depends on the communication strategies of online media, as well as on determining their health event reporting threshold, and how these features impact the sensitivity of EBS systems.

The three EBS systems included in this study monitor media in multiple languages, thus facilitating detection of local media news. A further increase in the number of available languages should enhance the sensitivity of EBS systems (Barboza et al., 2014). Our study also showed that the three EBS systems were complementary regarding scope (animal and public health), moderation (manual, semi‐automated, automated) and number of covered languages.

PADI‐web could retrieve COVID‐19‐related news through animal health‐related RSS feeds, thus proving its usefulness for the detection of information of relevance for public health risk assessors. From 275 COVID‐19‐related‐news items retrieved by PADI‐web, 54.5% (*n* = 150) were retrieved via syndromic RSS feeds, while the remaining 45.5% (*n* = 125) were retrieved via disease‐specific RSS (Table 1).

**Table 1:** Percentage (%) and number (*n*) of COVID‐19‐related news items retrieved by PADI‐web from 31 December 2019 to 26 January 2020

Link with COVID‐2019	Type of RSS feed		
	Disease‐specific	Syndromic	Total
Comparison with another disease	20.4% (*n* = 56)	11.3% (*n* = 31)	31.7% (*n* = 87)
Disease ruled out	17.8% (*n* = 49)	0.4% (*n* = 1)	18.2% (*n* = 50)
Aggregation with other disease outbreaks	6.2% (*n* = 17)	1.5% (*n* = 4)	7.7% (*n* = 21)
Coronaviruses in animals	‐	24.4% (*n* = 67)	24.4% (*n* = 67)
Market animals	‐	2.5% (*n* = 7)	2.5% (*n* = 7)
Avoid contact with animals	‐	0.7% (*n* = 2)	0.7% (*n* = 2)
Irrelevant keyword matches	0.4% (*n* = 1)	4.0% (*n* = 11)	4.4% (*n* = 12)
Unknown	0.7% (*n* = 2)	9.8% (*n* = 27)	10.5% (*n* = 29)
Total	45.5% (*n* = 125)	54.5% (*n* = 150)	100% (275)

Note

Each article is categorized by type of feed (disease‐related or syndromic) according to the link between the feed and COVID‐19.

Content‐wise, 31.7% (*n* = 87) of the news items compared COVID‐19 to five animal diseases (avian influenza, African swine fever, classical swine fever, West Nile virus and Rift Valley fever), 24.4% (*n* = 67) of the news items described the broad range of animal species sensible to coronaviruses, 18.2% (*n* = 50) reported that avian influenza was ruled out from possible causes of the outbreak, 7.7% (*n* = 21) described other ongoing outbreaks in addition to COVID‐19 (avian influenza, African swine fever, classical swine fever and foot‐and‐mouth disease), 2.5% (*n* = 7) referred to animal species present in Chinese markets as potential COVID‐19 sources, and 0.7% (*n* = 2) advised people to avoid contact with animals. Irrelevant keyword matches were found in 12 news items (e.g. finding a host keyword in the name of a source), and no link could be established between the RSS feed and the article for 29 other news items (10.5%).

The fact that disease‐specific RSS feeds contributed as much as syndromic RSS feeds to the detection of COVID‐19 news by PADI‐web was unexpected, thus highlighting the importance of combining both disease‐specific and syndromic feeds. Many of the news items detected by PADI‐web compared the magnitude and economic impact of COVID‐19 with regard to avian influenza and African swine fever outbreaks in China. Indeed, prior to COVID‐19 identification, the reported pneumonia‐like illness was compared to avian influenza zoonotic infections. Some news also presented a summary of several recent disease outbreaks in China, including African swine fever (which is not a zoonotic disease), thus explaining why they were detected by PADI‐web.

The ability of EBS tools to encompass a broad scope of health‐related topics through a limited number of queries (RSS feeds) is a major asset compared to formal sources. This capacity largely depends on the intrinsic features of online news in which outbreak‐related content is often bulked up with additional information, such as comparisons with previous disease outbreaks, thus increasing the probability of being detected by EBS tools. However, the probability of detection of an EID event might be higher for (actual or assumed) zoonotic diseases and countries with ongoing animal disease outbreaks. This is not a major shortcoming in practice.

### 3.2 News vocabulary

From the terms referring to either the virus or the disease, 18 terms were in the ‘pneumonia’ category, eight terms in the ‘mystery’ category, three terms in the ‘coronavirus’ category (one of them, ‘coronovirus’ being a misspelt form of ‘coronavirus’), and seven terms in the ‘technical’ category (Table 2).

**Table 2:** Terms used to describe SARS‐CoV‐2 and COVID‐19 in the corpus and their corresponding category after manual classification

Category	Terms
Pneumonia	pneumonia, respiratory outbreak, lung disease, respiratory tract illness, respiratory illness, respiratory infection, pneumonia‐like disease, upper‐respiratory illness, respiratory condition, lung infection, pneumonia‐like cases, pneumonia‐like illness, respiratory virus, lung virus, pneumonia‐like virus, pneumonia‐causing virus, pneumonia‐like virus.
Mystery	mystery, mysterious, unidentified, undocumented, disease x, unknown, abnormal, unexplained.
Technical	2019‐ncov, ncov, 2019 novel coronavirus, n‐cov2019, novel coronavirus 2019, ncov2019, cov2019.
Coronavirus	coronavirus, betacoronavirus, coronovirus

The wordclouds generated from the overall news contents mined over three consecutive periods are shown in Figure 1.

**Figure 2:**
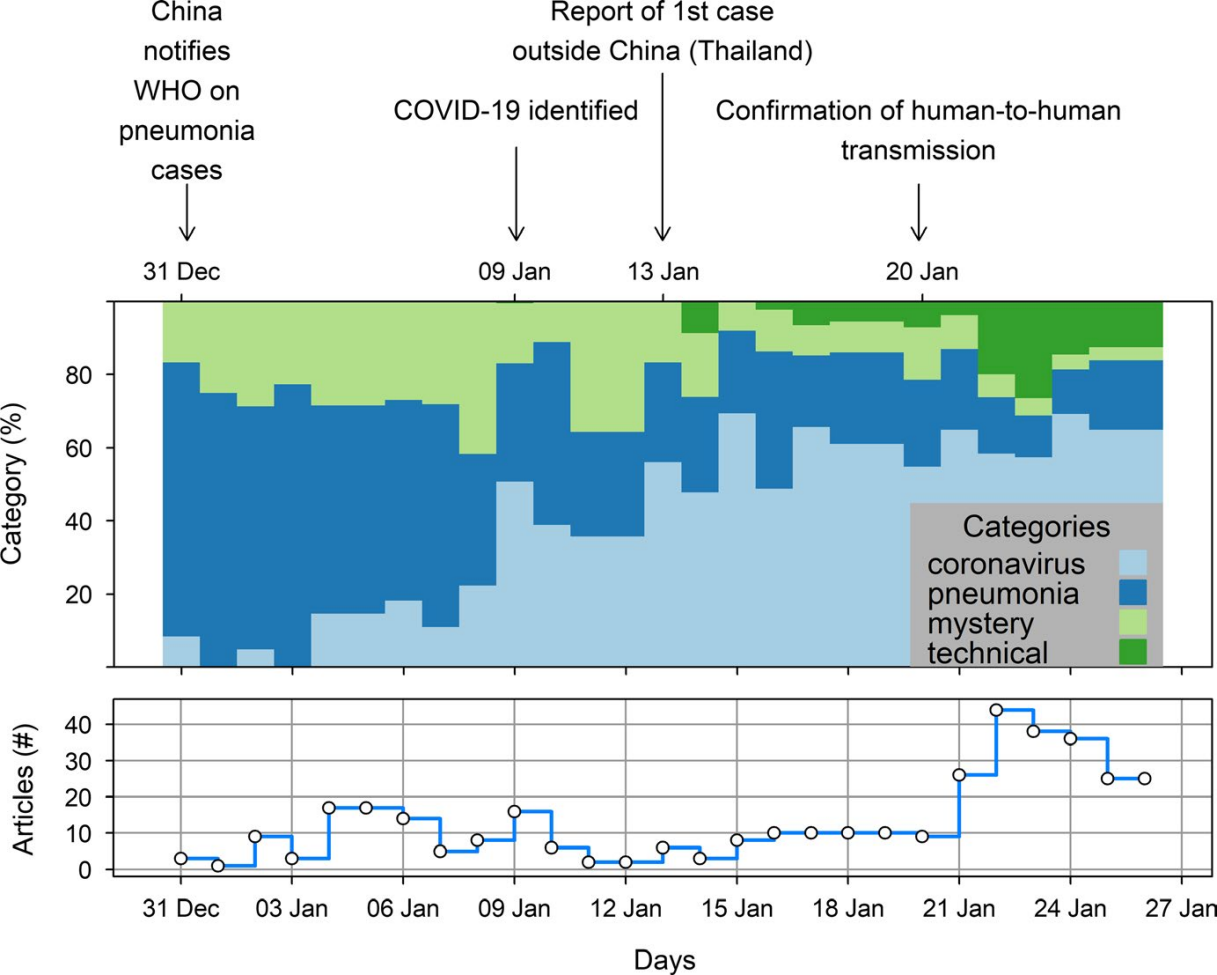
Frequency of the different categories used to describe COVID‐19 outbreaks (above) and stepped curve of the daily number of COVID‐19 news articles retrieved by PADI‐web (below), from 31 December 2019 to 26 January 2020. Daily counts for Saturday and Sunday are merged to account for weekday/weekend trends [Colour figure can be viewed at wileyonlinelibrary.com]

Before identification of the virus (31 December 2019 – 8 January 2020), 58.1% (*n* = 317) of the COVID‐19 terms were in the ‘pneumonia’ category, 29.1% (*n* = 159) in the ‘mystery’ category and 12.8% (*n* = 70) in the ‘coronavirus’ category. From the official identification of the virus to the first report of a case outside China (09 – 12 January 2020), 48.5% (*n* = 127) of the terms were in the ‘coronavirus’ category, 34.7% (*n* = 91) in the ‘pneumonia’ category, and 16.8% (*n* = 44) in the ‘mystery’ category. From this first report to the confirmation of human‐to‐human transmission (13–19 January 2020), 58.3% (*n* = 196) of the terms were in the ‘coronavirus’ category, 27.4% (*n* = 92) in the ‘pneumonia’ category, 11.3% (*n* = 38) in the ‘mystery’, category and 3.0% (*n* = 10) were in the ‘technical’ category. From the confirmation of human‐to‐human transmission to the end of the studied period 62.9% (*n* = 906) of the terms were in the ‘coronavirus’ category, 17.4% (*n* = 250) in the ‘technical’ category, 14.1% (*n* = 203) in the ‘pneumonia’ category, and 5.6% (*n* = 81) in the ‘mystery’ category (Figure 2).

The incorporation of terms semantically related to ‘unknown’ and ‘mysterious’ events into existing RSS feeds could enhance the detection and retrieval of relevant news. We suggest that this category of terms could boost the identification of classic epidemiological entities (e.g. disease, hosts, locations, dates) in news feeds.

Our results revealed that the vocabulary changed as the disease spread. EBS methods used to mine and analyse news from the web should thus be tailored to the different disease epidemiology stages.

With MERS‐CoV in 2014 and SARS in 2003, COVID‐19 is the third coronavirus outbreak emergence that has occurred over the past two decades, thereby highlighting the need to closely monitor the emergence of pneumonia‐like illnesses using existing EBS systems. Our results revealed the complementarity of the existing systems and underlined the need for collaborative development. Pooling veterinary and public health information resources seems crucial to improve early detection of unknown diseases in a One Health context. Our future work will focus on identifying the most relevant keywords for rapid detection of unknown threats, in collaboration with experts of other EBS systems. Moreover, EBS tools may be used in a broader setting, such as monitoring the implementation of protective and control measures.

Efforts invested in improving the timeliness and sensitivity of EBS systems make sense if their outputs (EID event alerts) are formatted, supervised and interpreted by epidemiologists in collaboration with disease experts and reference laboratories. Most importantly, EID event alerts should feed the risk assessment process to ensure early mitigation of EID events by the health managers and decision‐makers.

## CONFLICTS OF INTEREST

The authors declare no conflicts of interest.

## ETHICAL APPROVAL

The authors confirm compliance with the ethical policies of the journal, as noted on the journal's author guidelines page. No ethical approval was required because this study did not involve any experimental protocol on humans or animals, and only open source online data were used.

## Data Availability

The data that support the findings of this study are available for free download in the CIRAD Dataverse at https://doi.org/10.18167/DVN1/MSLEFC.

## ENDNOTES

^1^
Using the F‐TFIDF‐C measure (Lossio‐Ventura et al., 2014).

^2^

https://promedmail.org/promed‐post/?id=6864153
